# High prevalence of femoral and tibial torsional abnormalities in female patients with anterior knee pain resistant to conservative treatment: A CT‐based study

**DOI:** 10.1002/jeo2.70446

**Published:** 2025-09-27

**Authors:** Vicente Sanchis‐Alfonso, Cristina Ramírez‐Fuentes, Erik Montesinos‐Berry, Julio Doménech‐Fernández

**Affiliations:** ^1^ Department of Orthopaedic Surgery Hospital Arnau de Vilanova Valencia Spain; ^2^ Department of Radiology Hospital Universitario y Politécnico La Fe Valencia Spain; ^3^ ArthroCentre Orthopaedics Riaz & Clinique CIC Riviera Montreux Switzerland

**Keywords:** acetabular version, anterior knee pain, external tibial torsion, femoral anteversion, patellofemoral pain

## Abstract

**Purpose:**

To assess acetabular version (AV) and to determine the prevalence of femoral and tibial torsional abnormalities in female patients with anterior knee pain (AKP) unresponsive to conservative treatment. The study also aimed to evaluate the prevalence of combined abnormalities.

**Methods:**

Seventy‐three AKP female patients resistant to conservative treatment evaluated between 2013 and 2024 were included. Torsional computed tomography (CT) of the lower limbs was performed on all cases, resulting in the evaluation of 146 limbs. Femoral anteversion (FAV) was measured using Murphy's method, external tibial torsion (ETT) using Jend's technique and AV using the method described by Tönnis and Heinecke.

**Results:**

Torsional abnormalities were highly prevalent. Only 13.0% of limbs had normal ETT, 43.2% showed moderate abnormalities and 43.8% severe abnormalities. FAV was within normal limits in 48.6% of cases, while 26.7% had moderate anteversion, 22.6% severe anteversion and 2.1% retroversion. AV was normal in 82.9% of limbs, with 13.7% showing retroversion and 3.4% anteversion. Only 4.1% of patients exhibited normal across all three parameters, while 8.2% presented combined abnormalities. The most common paired association was observed for ETT and FAV (35.6%). Chi‐squared analyses did not reveal statistically significant associations among the degrees of deformity. Pearson correlation analysis showed a weak but statistically significant correlation between ETT and FAV (*r* = 0.176, *p* = 0.034).

**Conclusion:**

In young female patients with AKP unresponsive to conservative treatment, assessment of femoral and tibial torsion using CT imaging should be systematically considered. This approach is essential for guiding clinical decision‐making. The range of possible torsional abnormalities highlights the importance of patient‐specific evaluation.

**Level of Evidence:**

Level IV.

**Abbreviations**
AKP: anterior knee pain
AV: acetabular version
CT: computed tomography
ETT: external tibial torsion
FAV: femoral anteversion
MRI: magnetic resonance imaging

## INTRODUCTION

Patellofemoral joint pathology can be categorized into three main groups: chronic lateral patellar instability, degenerative conditions and patellofemoral pain or anterior knee pain (AKP) in young patients [16]. While the treatment for the first two clinical entities is well‐established in daily practice and generally yields good clinical outcomes, the same cannot be said for the management of AKP patients. Traditionally, it has been stated that AKP should be treated conservatively and that surgery should be reserved for the few cases in which physical therapy fails. However, even with appropriate conservative treatment, satisfactory results are achieved in only 60% of cases [2]. This raises a crucial question: If conservative treatment is so effective, how is it possible that 40% of our patients with AKP do not improve? The answer seems evident; we are not making an accurate diagnosis [15]. In other words, we may be overlooking potential causes of AKP that we are not evaluating and that are not amenable to conservative treatment [5, 6, 12, 15, 18, 23, 25, 26]. Therefore, structural rather than functional factors are likely responsible for the failure of conservative management, making them unsuitable for physiotherapy‐based approaches. One such potential factor is torsional abnormality [5, 6, 12, 15, 18, 23, 25, 26]. To date, no studies have specifically analyzed the torsional profile of AKP patients who have failed conservative treatment.

The objectives of our study are to assess acetabular version (AV) and to determine the prevalence of femoral and tibial torsional abnormalities in female AKP patients unresponsive to an adequate conservative treatment. This study also aimed to evaluate the prevalence of combined abnormalities. Our main working hypothesis is that torsional abnormalities are a more frequent cause of AKP than previously recognized. Our second working hypothesis is that the AV is normal in the vast majority of AKP patients with femoral maltorsion.

## MATERIALS AND METHODS

### Participants and study design

This study was approved by our institutional review board (CEIm Hospital Arnau de Vilanova; Protocol #PI 21_2024). From January 2013 to December 2024, a total of 203 consecutive AKP patients were referred to our Knee Unit after failing to respond to appropriate conservative treatment. January 2013 marks the point when the first author of this study began systematically considering torsional abnormalities in the evaluation and management of AKP patients. For patients who did not respond to conservative treatment, the standard radiographic workup included weight‐bearing whole‐limb anteroposterior radiographs, computed tomography (CT) scans to assess torsional abnormalities and magnetic resonance imaging (MRI). All patients were evaluated and treated by the first author.

**Figure 1:**
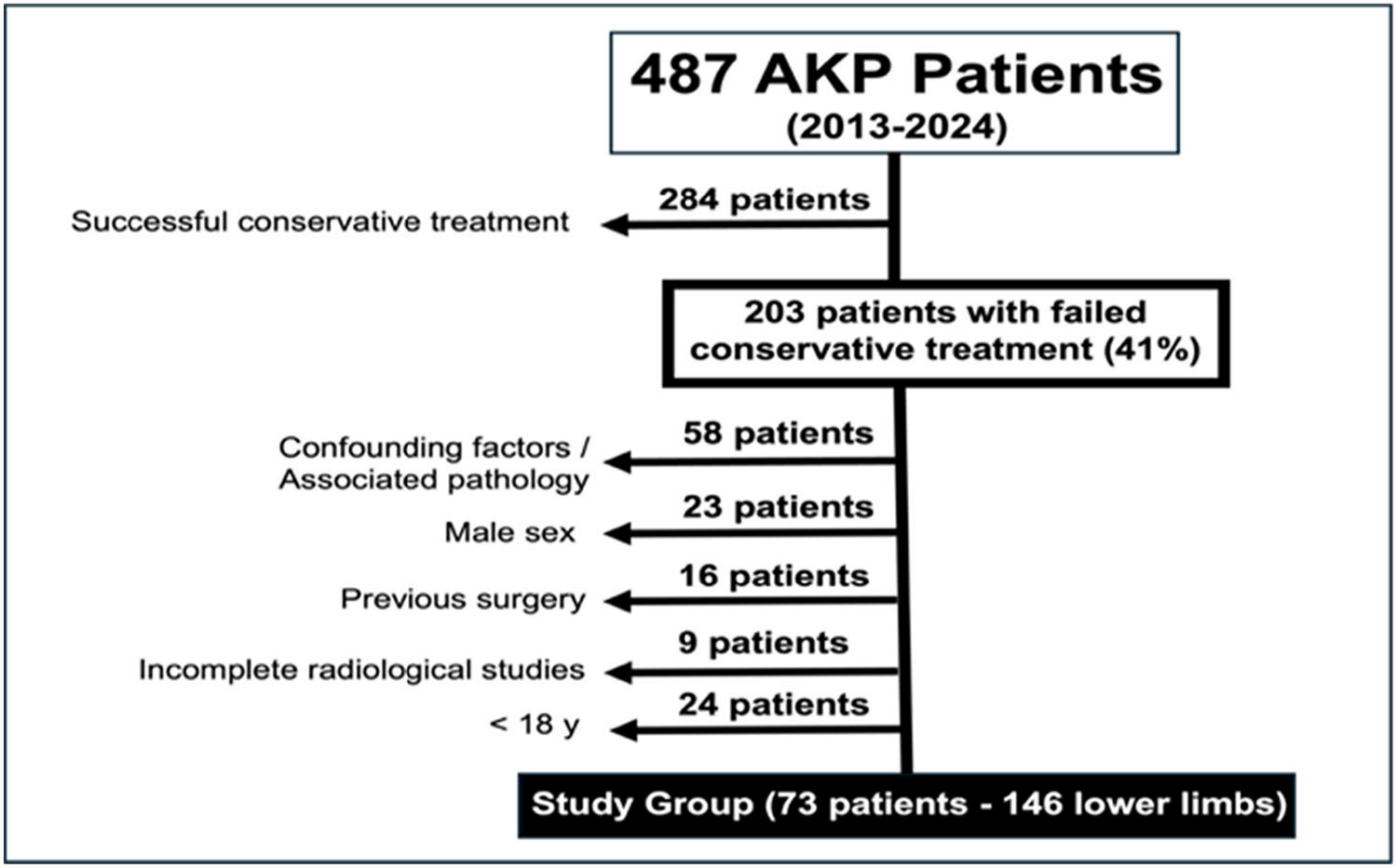
Flowchart of patient enrolment. Inclusion and exclusion criteria for the study group. AKP, anterior knee pain.

From the initial 203 patients, 130 were excluded for the following reasons. Patients with associated lesions (osteoarthritis, severe chondropathy, meniscal or ligamentous injury, and trochlear dysplasia Grade B, C and D) that provoke knee pain, or with confounding anatomical factors known to contribute to AKP, as identified in the literature, including, tibial tuberosity–trochlear groove distance >20 mm on CT scan, patella alta (Index of Caton–Deschamps > 1.4), trochlear dysplasia Grade B, C and D according to Dejour classification, and patellar tilt >20° (58 patients), male patients (23 patients), <18 years old (24 patients), history of prior femoral, tibial or tibial tubercle surgery (16 patients) and incomplete radiological studies (9 patients). This left a total of 73 female patients (146 lower limbs) for inclusion in this study. The flowchart of patient enrolment is shown in Figure 1. Only female patients were included in this study since AKP predominantly affects women. Focusing on women in AKP research ensures relevance to the most affected population while accounting for gender‐specific anatomical, biomechanical and hormonal factors. This approach enhances the validity of findings. All torsional CT studies were retrospectively collected to evaluate AV and femoral and tibial torsion. All CT scans were performed and assessed by the same musculoskeletal radiologist (C.R.‐F.). The measurement methods employed are routinely used in our institution and have shown very good interobserver reliability: ICC of 0.916 for tibial torsion [11], ICC of 0.960 for femoral anteversion (FAV) [3] and ICC of 0.75 for AV [13]. The CT scans were conducted strictly for clinical reasons as part of the diagnostic protocol of our knee unit. No a priori sample size was calculated because this study included all patients with CT torsional studies at our institution.

### CT protocol—evaluated study variables

CT images were acquired using a high‐spatial resolution 256‐detector row CT scanner (Brilliance iCT; Philips). Patients were positioned supine with the hip and knee fully extended and the feet in 15° of external rotation. Three separate CT scans were obtained for each patient: a hip scan—from the upper edge of the femoral heads to immediately distal to the lesser trochanters, a knee scan—from the upper edge of the patella to immediately distal to the tibial tuberosity; and an ankle scan—including the tibial plafond and both malleoli. The raw data sets acquired were 64 × 0.625 mm collimation, 0.5 s rotation time, 0.9 mm slice reconstruction thickness, 0.45 pitch, 120 kV and automated mAs control. Torsional measurements were performed manually using the imaging tools on the Advantage Workstation 4.5 Software (GE HealthCare) integrated in the picture archiving and communication system. All images in the present study have been previously anonymized. Once anonymization was carried out, a musculoskeletal radiologist with over 15 years in practice (C.R.‐F.) performed all measurements and included them in the database.

**Figure 2:**
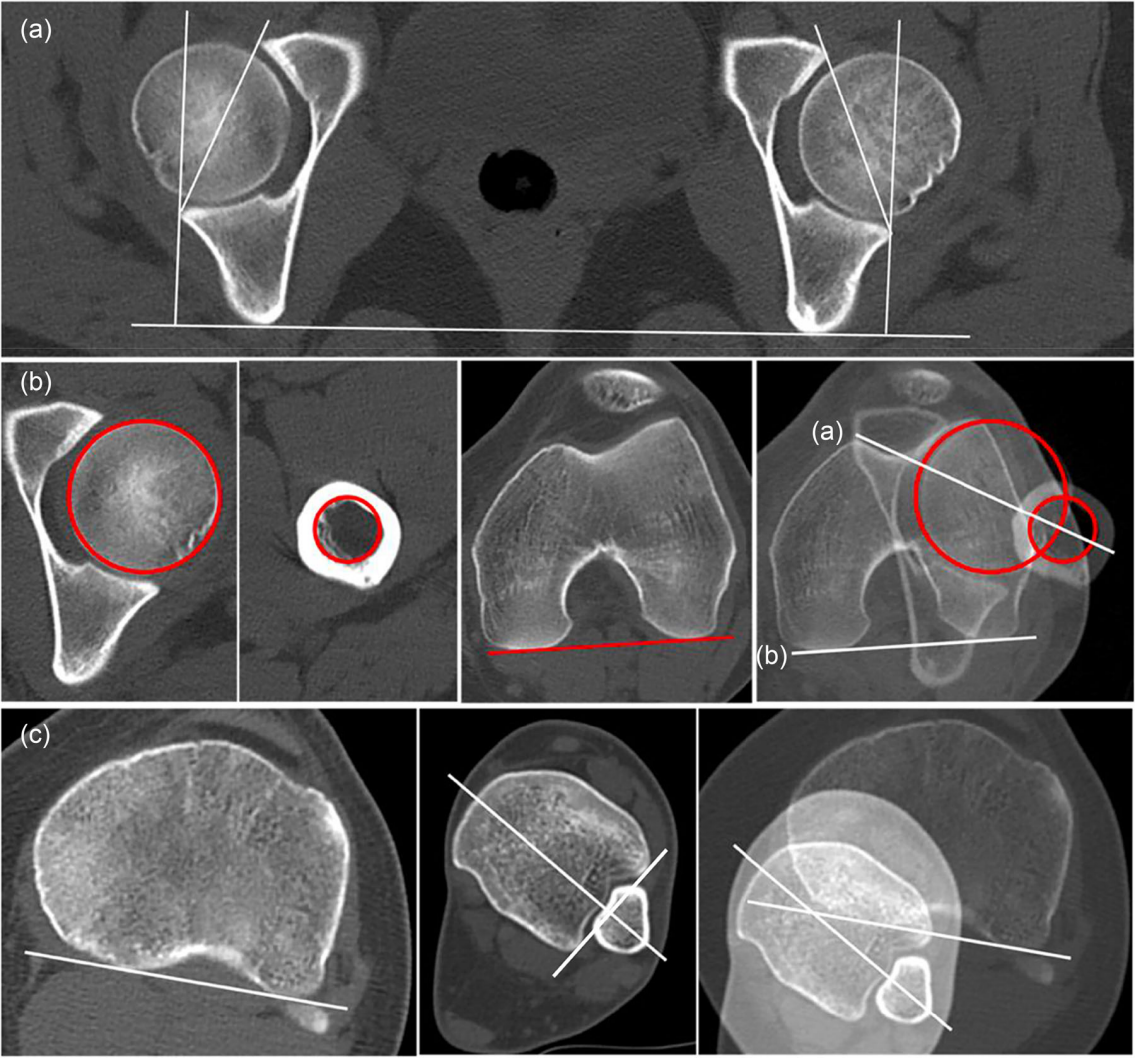
(a) Acetabular version measurement: after selecting the transverse slice at the centre of the head (where the head appears largest), calculate the angle between a line perpendicular to the horizontal line connecting the posterior margins of the ischia and a line connecting the anterior and posterior acetabular rims on the same slice. (b) Femoral torsion measurement: three transverse images were selected, one at the centre of the femoral head, one at the femoral shaft immediately distal to the lesser trochanter and one at the posterior intercondylar line. One circle was placed on the centre of the femoral head, and another was placed on the femoral shaft immediately distal to the lesser trochanter. Murphy's angle was obtained from the posterior intercondylar line and the line connecting the centre of the femoral head to the centre of the femoral shaft distal to the lesser trochanter. (c) Tibial torsion measurement: two transverse sections are selected, one at the proximal level of the tibial plateau and one at the level of the distal tibiofibular syndesmosis. The posterior condylar axis (tangent to the more proximal tibial plateau, where the posterior condylar notch was clearly recognized) constituted the proximal reference line. The line joining the most protruding part of the lateral and medial malleolus, perpendicular to the fibular notch of the tibia, is the distal line of reference. Tibial torsion is the angle formed between the two lines.

Three variables were then assessed: AV, femoral torsion and tibial torsion (Figure 2). AV was measured according to the Tonnis and Heinecke method [27] and defined as the angle between a sagittal line and a line connecting the anterior and posterior acetabular rim at the centre of the femoral head. A normal AV was defined from 10° to 25° [10]. For the FAV measurement, Murphy's method was used [14]. For this, three transverse images were selected in each limb: the centre of the femoral shaft immediately distal to the lesser trochanter, the centre of the femoral head and the posterior intercondylar line. One circle was placed at the centre of the femoral head, and another on the femoral shaft. The Murphy's angle was obtained from two lines, the posterior intercondylar line (Line B) along with the line connecting the centre of the femoral head and the centre of the femoral shaft immediately distal to the lesser trochanter (Line A) (Figure 2). Normal femoral version was considered to be 10° to 25° [10]. Severely decreased femoral version was defined as femoral version <0°, moderately decreased femoral version was defined as femoral version between 0° and 10°, moderately increased femoral version (25–35°) and severely increased femoral version (>35°) [10]. Total tibial torsion was assessed using a technique modified from Jend et al. [9]. The posterior condylar axis (tangent to the more proximal tibial plateau, where the posterior condylar notch was clearly recognized) constituted the proximal reference line. The distal reference line was formed by joining the most protruding part of the lateral and medial malleolus, perpendicular to the fibular notch of the tibia. Patients were classified into three groups for our study: normal external tibial torsion (ETT): ≤30°, moderate ETT: 31–40° and severe ETT: >40°.

### Statistical analysis

Descriptive statistics were used to determine the prevalence of individual and combined torsional abnormalities. For continuous variables (ETT, FAV and AV), Pearson correlation coefficients were calculated to assess the relationships between measurements. For categorical analyses, torsional measurements were classified according to established clinical thresholds. The prevalence of individual abnormalities was calculated as a percentage of the total sample. Combined abnormalities were assessed by calculating the percentage of patients presenting simultaneous abnormalities in all three measurements.

Chi‐squared tests of independence and Cramer's *V* coefficients were performed to analyze the associations between categorical variables in contingency tables. Three separate chi‐squared tests were conducted: ETT vs. FAV, ETT vs. AV and FAV vs. AV. The chi‐squared test statistic, degrees of freedom and *p* values were calculated using the chi2_contingency function from scipy.stats. Statistical significance was set at *p* < 0.05. All statistical analyses were performed using Python (version 3.11) with scipy.stats, pandas and numpy libraries.

## RESULTS

Details on patient demographics extracted from the hospital's database are reported in Table 1. A total of 146 lower limbs were analyzed. The individual abnormalities were categorized based on established clinical thresholds. For tibial torsion, 86.9% of patients showed increased ETT, and only 13.0% were classified as normal. In FAV, 51.4% of patients demonstrated abnormal values (26.7% moderate anteversion, 22.6% severe anteversion and 2.1% retroversion), while 48.6% were normal. AV was abnormal in 17.1% of patients (13.7% retroversion and 3.4% anteversion), with the majority (82.9%) showing normal values (Table 2).

**Table 1:** Demographic and clinical characteristics of the patient cohort.

Patients (*n*)	73 (146 lower limbs)
Age (years)	23.3 ± 6.4 (18–47)
Limb R/L	73/73
BMI	21 ± 4.5 (18.3–33.9)

Abbreviation: BMI, body mass index.

**Table 2:** Prevalence of femoral and tibial torsion and acetabular version in AKP patients refractory to conservative treatment.

	Degrees	*N* (%)
Acetabular version	16.1 ± 10.5	146 (100%)
Normal (10–25°)	17.5 ± 4.6	121 (83%)
Retroversion (<10°)	4.9 ± 3.5	20 (13.7%)
Anteversion (>25°)	27.6 ± 1.5	5 (3.4%)
Femoral torsion	26.5 ± 10.5	146 (100%)
Normal (10–25°)	18.5 ± 4.4	71 (48.6%)
Retroversion (<10°)	5.7 ± 3.5	3 (2.1%)
Anteversion (>25°)	35.3 ± 6.8	72 (49.3%)
Moderate (26–35°)	30.1 ± 2.7	39 (26.7%)
Severe (>35°)	41.5 ± 4.7	33 (22.6%)
Tibial torsion	39.5 ± 8.7	146 (100%)
Normal (≤30°)	26.5 ± 3.8	19 (13%)
External tibial torsion	41.4 ± 7.5	127 (86.9%)
Moderate (31–40°)	35.9 ± 2.7	63 (43.2%)
Severe (>40°)	46.9 ± 6.7	64 (43.8%)

Abbreviation: AKP, anterior knee pain.

When evaluating the overall torsional profile, only 4.1% of patients had entirely normal torsional measurements across all three parameters. Isolated abnormalities were relatively frequent, but the combination of deformities was less common. The combination of ETT and FAV was the most common paired deformity (35.6%) (Table 3). When assessing combined abnormalities across all three parameters (ETT, FAV and AV), only 8.2% of patients exhibited concurrent deformities.

**Table 3:** Contingency table of tibial torsion vs femoral torsion expressing the cases and percentage in each category of deformity.

	Femoral torsion			
	Anteversion	Normal	Retroversion	Total
Tibial torsion				
Normal	1 (0.7%)	16 (11.0%)	2 (1.4%)	19 (13.0%)
Moderate	2 (1.4%)	53 (36.3%)	8 (5.5%)	63 (43.2%)
Severe	2 (1.4%)	52 (35.6%)	10 (6.8%)	64 (43.8%)
Total	5 (3.4%)	121 (82.9%)	20 (13.7%)	146 (100%)

Contingency table analyses were performed to assess whether there were associations between the graded abnormalities. Chi‐squared tests and Cramer's *V* coefficients were calculated to assess the associations between torsional abnormalities. The analysis revealed no statistically significant associations between any pair of measurements, although there was a trend toward significance in the relationship between tibial torsion and FAV (*χ* ^2^ = 10.806, df = 6, *p *= 0.0946, Cramer's *V* = 0.192). The association between tibial torsion and AV was notably weak (*χ* ^2^ = 0.611, df = 4, *p* = 0.961, Cramer's *V* = 0.046), as was the relationship between FAV and AV (*χ* ^2^ = 4.700, df = 6, *p* = 0.582, Cramer's *V* = 0.127). The Cramer's *V* values, all below 0.2, indicate weak associations among all paired comparisons, suggesting that these torsional abnormalities generally occur independently rather than in predictable combinations.

A further analysis using Pearson correlation coefficients was conducted among the continuous measurements of ETT, FAV and AV. A very weak but statistically significant positive correlation was found between ETT and FAV (*r* = 0.176, *p* = 0.034). The correlations between ETT and AV (*r* = −0.031, *p* = 0.713) and between FAV and AV (*r* = 0.122, *p* = 0.141) were not statistically significant. These findings suggest that while there is a slight tendency for ETT to increase with FAV, the relationships between these torsional measurements are generally very weak.

## DISCUSSION

The most important finding of this study is that femoral and/or tibial torsional abnormalities are highly prevalent in female AKP patients who are recalcitrant to conservative treatment. However, the lower limb torsional profile in AKP patients is frequently not assessed in routine clinical practice. Notably, while tibial and femoral torsional abnormalities were common (86.9% and 51.4% respectively), AV abnormalities showed a markedly lower prevalence (17.1%), suggesting that AV should not be as relevant in the AKP patient as femoral and tibial torsion.

Overall, while isolated torsional abnormalities are common in our cohort, a significant association among the deformity grades, except for a borderline trend between ETT and FAV, was not found. Moreover, the strength of these associations is weak. Only a small proportion (8.2%) of patients have combined abnormalities across all three parameters. These findings suggest that although distinct torsional abnormalities are prevalent, they largely occur independently rather than in a consistent combined pattern. This highlights the importance of individualized evaluation and treatment planning for AKP patients.

One of the main challenges of this study was selecting an appropriate control group for comparison of AV and femoral and/or tibial torsion prevalence in AKP patients resistant to conservative treatment. Using healthy women with no musculoskeletal symptoms in the lower limbs as a control group posed the ethical concern of exposing them to radiation from CT scans. However, a recent study using MRI instead of CT reported similar FAV values [28]. In the future, MRI could enable FAV measurement without the drawback of radiation exposure. Another option we considered during study design was using the asymptomatic contralateral limb in unilateral cases as a control. However, in most cases, the symptoms are bilateral, although with varying degrees of involvement. And in the few unilateral cases, the asymptomatic side often becomes symptomatic. Moreover, clinical experience suggests that when one limb is operated on and the symptoms leading to a derotational osteotomy resolve, the previously asymptomatic or mildly symptomatic contralateral limb frequently becomes symptomatic [17]. This occurs because the patient now has a reference point for comparison after experiencing pain relief in the treated limb [17]. Therefore, we concluded that the asymptomatic contralateral limb is not a reliable control group. Consequently, we chose to compare our results with the values considered normal in the medical literature [10].

Another problem of this study was selecting the appropriate method for assessing FAV. Multiple techniques exist for measuring FAV, and the results can vary by up to 20° depending on the method used [21, 25]. Therefore, when discussing FAV values, it is essential to specify the measurement technique employed. In our daily clinical practice, we use the method described by Murphy et al. [14]. We selected Murphy's method because it closely reflects anatomical reality and has good reproducibility [13, 19, 20]. Moreover, the method described by Murphy et al. overestimates femoral version by only 3.5°, a relatively small discrepancy compared to other CT‐based techniques [24]. According to Meier et al. [13], normal FAV ranges from 10° to 25°, as measured using Murphy's method. In our series, 45.3% of patients had pathological FAV, with severe cases accounting for 20.5% of these. Interestingly, the mean femoral torsion value was not excessively high (26.53 ± 10.48°), likely because cases of retroversion (2.7%) partially neutralized the overall mean. Regarding AV, 83% of cases fell within the normal range, and in the 17% with elevated or decreased values, but deviations from the limit of normality were no greater than 3° in most cases, a difference that is not clinically significant. The standard deviation was considerably larger for femoral torsion (26.53 ± 10.48°) than for AV (16.17 ± 6.38°), indicating a greater range of variation in femoral torsion values. Based on these findings, we can conclude that AV does not play a significant role in AKP, whereas pathological FAV does.

The third challenge of this study was determining the threshold for pathological tibial torsion. Numerous studies have sought to define the normal range of ETT in young, asymptomatic individuals [7, 8, 22, 23, 29]. ETT has been evaluated through various methods, including clinical assessments, anatomical studies and imaging modalities such as ultrasound, MRI and CT scans [7, 8, 22, 23, 29]. Based on anthropometric data, cadaveric analyses and imaging studies, the normal range for ETT in individuals of European origin is generally considered to be between 24° and 30° [23]. However, significant variability exists due to differences in patient demographics, age and racial groups. Currently, there is no universally accepted threshold distinguishing normal from pathological ETT. Studies on this topic report varying cut‐off points, making it impractical to define study groups solely based on these thresholds. Instead, our study employed cut‐off values associated with surgical indications for tibial derotational osteotomy to categorize patients. Most published surgical series accept a threshold of 30° (measured using the bimalleolar method) for surgical intervention, as osteotomy in these cases has shown improvements in both clinical symptoms and objective measurements [1, 5, 6, 12]. However, some authors advocate for surgery only when ETT exceeds 40°, which could serve as an alternative threshold [4]. The three groups used in our study are based on the above‐mentioned criteria.

### Clinical implications

This study has significant clinical implications. Based on our findings, torsional profile evaluation using CT should be performed in all AKP patients in whom an appropriate conservative 6‐month treatment has failed. Moreover, the relative independence of these abnormalities emphasizes the importance of comprehensive evaluation of all torsional parameters, as the presence of one abnormality does not reliably predict others. The presence of combined deformities underscores the necessity for patient‐specific evaluation, meaning that each patient requires a carefully tailored assessment. Since femoral and tibial torsion are independent, unrelated deformities, when we have a torsional abnormality of the femur and tibia, the two deformities should be corrected independently, as long as both are pathological. We cannot expect the correction of one to compensate for the problems caused by the other. Finally, the low prevalence of AV abnormalities suggests that this parameter may be less critical in the evaluation of AKP patients. Although AKP is linked to torsional abnormalities, this does not mean that the presence of a torsional abnormality automatically leads to its surgical correction. We should always exhaust a tailored conservative treatment before osteotomy surgery.

### Study limitations

One limitation of our study is the absence of a control group, as we relied on normal values from the medical literature. Furthermore, our sample includes only females, while the control group from the medical literature includes males and females. Our study is based on a consecutive series of patients, but it represents a highly selective cohort referred to a specialized patellofemoral pathology unit. As a result, the severity of cases seen in our unit may be greater than that of the general AKP population, which could explain the higher prevalence of femoral and/or tibial torsional abnormalities in our cohort. This introduces potential selection bias. Therefore, our findings may not be directly generalizable to the broader AKP patient population. Additionally, the definition of pathological FAV or ETT varies across the medical literature, meaning that our study results could differ depending on the cut‐off values used. Finally, to minimize bias and given that AKP predominantly affects women, we exclusively evaluated female patients. Therefore, our conclusions apply only to female patients.

## CONCLUSION

In young female patients with AKP unresponsive to conservative treatment, assessment of femoral and tibial torsion using CT imaging should be systematically considered. This approach is essential for guiding clinical decision‐making. The range of possible torsional abnormalities highlights the importance of patient‐specific evaluation.

## AUTHOR CONTRIBUTIONS

*Project coordinator, designing the study, writing the manuscript, evaluating the radiographs, collecting and analyzing the data*: Vicente Sanchis‐Alfonso. *Writing the manuscript, evaluating the radiographs and collecting data*: Cristina Ramírez‐Fuentes. *Writing the manuscript*: Erik Montesinos‐Berry. *Writing the manuscript and analyzing the data*: Julio Doménech‐Fernández.

## CONFLICT OF INTEREST STATEMENT

The authors declare no conflicts of interest.

## ETHICS STATEMENT

CEIm Hospital Arnau de Vilanova, Valencia, Spain #PI 21_2024.

## Data Availability

The data that support the findings of this study are available from the corresponding author upon reasonable request.
